# Head and Neck Sarcoma Assessor (HaNSA) for treatment decisions using real-world data

**DOI:** 10.1016/j.esmorw.2024.100069

**Published:** 2024-09-05

**Authors:** M.Y.S. See, J.J.N. Goh, C.E. Low, C.E. Yau, W.S. Ong, R.X. Wong, N.F. Mohamed Noor, M.H.B.H. Mohamed, J.T. Suha, A.N.H. Sairi, W.L. Goh, X.Y. Woo, V.S. Yang

**Affiliations:** 1 Lee Kong Chian School of Medicine, Nanyang Technological University, Singapore; 2 Bioinformatics Institute (BII), Agency for Science, Technology and Research (A∗STAR), Singapore; 3 Yong Loo Lin School of Medicine, National University of Singapore, Singapore; 4 Division of Clinical Trials and Epidemiological Sciences, National Cancer Centre Singapore, Singapore; 5 Division of Radiation Oncology, National Cancer Centre Singapore, Singapore; 6 Division of Medical Oncology, National Cancer Centre Singapore, Singapore; 7 Translational Precision Oncology Laboratory, Institute of Molecular and Cell Biology, A∗STAR, Singapore; 8 Oncology Academic Clinical Program, Duke-NUS Medical School, Singapore

**Keywords:** head and neck, sarcoma, Asia, survival, real-world, parametric model

## Abstract

**Background:**

Head and neck sarcomas (HNS) are rare and diverse cancers with distinct biology, unique treatment constraints and poor survival outcomes. Furthermore, HNS are understudied in Asians, and prospective clinical trials are untenable. To better understand HNS and improve treatment, real-world studies in Asians with accurate histological typing are thus needed.

**Materials and methods:**

A retrospective cohort study of patients with histologically confirmed sarcoma diagnosis in the head and neck region between 1985 and 2023 was carried out at the National Cancer Centre Singapore. Multivariate Cox regression was used to analyse risk factors for overall survival (OS), and parametric time-to-event modelling was used to develop a prognostic calculator.

**Results:**

A total of 275 patients were analysed. The 5-year OS was 43.2% (95% confidence interval 36.2% to 51.6%). Among demographic risk factors, a high incidence of radiotherapy-associated sarcomas in the population at 11.3% placed the population at higher risk for aggressive disease (decreased treatment response and poorer prognosis). With interventions, microscopically negative (R0) surgical resection margins were significantly associated with improved OS. Parametric time-to-event simulations suggested microscopically positive (R1) resections to also be beneficial for OS in locally advanced tumours and nonaggressive sarcoma histology, and improved greatly alongside high-dose radiotherapy.

**Conclusion:**

We present the largest Asian HNS cohort, with diverse subtypes and disease extent. Our analysis highlights poor outcomes from a higher incidence of radiotherapy-associated disease, showing the challenging landscape of HNS in Asia. Through our prognostic calculator, we demonstrate how meaningfully curated real-world data in a rare disease entity can be used for the prediction of OS in individual patients with specific treatment approaches.

## Highlights

• Age, disease extent and radiotherapy-induced sarcomas predict OS.
• Only surgical resection margins were significantly associated with improved OS.
• Radiotherapy dose and chemotherapy use were not associated with OS.
• Our parametric model predicts OS based on patient, disease and key treatment factors.
• This prediction tool was developed using the largest published Asian cohort to date.

## Introduction

Cancer outcomes have improved overall in the past five decades, but this development is not shared with rare cancers, despite them accounting for up to 25% of all cancers.^1^^,^^2^ In particular, head and neck sarcomas (HNS) are not only rare, but reside in an anatomically challenging area for surgery.^3^ HNS comprise ∼1% of all head and neck malignancies and accounts for 5%-10% of all sarcomas, yet patients suffer much poorer outcomes relative to extremity sarcomas, with up to 29% lower survival rates.^4^, ^5^, ^6^, ^7^, ^8^, ^9^, ^10^, ^11^, ^12^, ^13^, ^14^, ^15^, ^16^ The sparse numbers of patients captured on record of most centres make the conduct of clinical trials and developing evidence-based best practices difficult. There is thus a need for real-world data, including that of patients not enrolled in clinical trials, to develop better evidence-based practices for HNS.

At present, only two large-scale cohort studies on HNS have been carried out.^7^^,^^11^ These studies primarily consist of Caucasian patients. Asian cohorts are thus not well represented in existing HNS studies.

Prognostic models of sarcoma such as Sarculator^17^ and PERSARC^18^ based on real-world data have garnered significant attention in recent years. However, these calculators have neither included HNS, nor included a significant Asian population, and did not assess the utility of multiple therapeutic interventions in their studies. Recent published HNS Asian cohort studies were limited to only South Korean (Park et al.^8^), or Han Chinese (Chen et al.^19^) ethnicities. Other Asian ethnicities have not been captured. Furthermore, real-world data are often unstructured, without clearly defined treatment groups for comparison. Thus, developing a prognostic model based on Asian HNS patients is essential to better understand and improve HNS outcomes and guide treatment in these patients.

The objective of this study was to characterise prognostic factors and develop a prognostic prediction tool incorporating key treatment modalities for Asian HNS patients. With this tool, we hope to improve management and outcomes for patients with HNS and spur the global advancement of our collective knowledge regarding HNS. This approach may lay the foundation for similar work in other rare cancers that are not amenable for the conduct of large-scale prospective studies or clinical trials.

## Materials and methods

### Patient inclusion and exclusion

This was a retrospective cohort study of HNS patients treated at the National Cancer Centre Singapore (NCCS) from 1985 to 2023. NCCS is Singapore’s largest cancer centre with the vast majority of local HNS catchment, and a major regional quaternary cancer centre managing HNS in the Southeast Asian region. Eligible patients were identified from an electronic database of sarcoma patients in NCCS. Inclusion criteria were the presence of a histologically confirmed sarcoma diagnosis, with the primary lesion originating in the anatomical region above the clavicles and a known diagnosis date. Exclusion criteria included sarcoma located within the cranial vault or spinal column, and patients without a subsequent follow-up visit after their diagnosis. This study was approved by the SingHealth Central Institutional Review Board (2018/3065).

### Outcomes

Our study investigated overall survival (OS) and progression-free survival (PFS). OS was defined as the duration from diagnosis until death as reported in the national death registry. PFS was defined as the duration from diagnosis until disease recurrence, progression or death, whichever occurred first. Patients without an event of interest in OS and PFS were censored at their last follow-up or at the 7-year mark, whichever occurred earlier. A 7-year right censoring was applied after an assessment of the median follow-up duration and given that <10% of patients had a >7-year follow-up duration.

### Covariates

Patient characteristics such as demographics, histology, disease extent and type of interventions received were extracted from electronic clinical case notes (Supplementary Table S1, available at https://doi.org/10.1016/j.esmorw.2024.100069). Histological classification of HNS was cross-checked by at least two subspecialist sarcoma pathologists. All past medical history and records were reviewed for possible radiotherapy-associated disease, and all patients were assessed clinically and staged radiologically for metastatic disease at diagnosis and as indicated in subsequent follow-up visits.

### Statistical methods

Initial dataset preparation, cleaning and statistical analysis were carried out using R (v 4.1.3.) packages tidyverse, lubridate, ggsurvfit and survival for survival analysis, and MASS for stepwise model building.

To build the prognostic prediction tool, feature selection was first carried out by removing variables that had ≥50% missing data. Among variables that were deemed highly correlated, variables that had more granularity to stratify patients, had more complete data or was more encompassing of the other categories were selected for model fitting (Supplementary Table S2, available at https://doi.org/10.1016/j.esmorw.2024.100069). This was done to prevent multicollinearity between variables in the fitted model. Next, univariate Cox proportional hazard (coxph) regression was carried out on each selected feature. Then, multivariable coxph models were built using a stepwise approach on features with *P* value < 0.05 on univariable coxph analysis. Histology, resection margins, radiotherapy dose and chemotherapy use were included in the multivariable model regardless of their statistical significance on univariable analysis, as our intention is to evaluate these interventions on different sarcoma subtypes. Proportional hazard assumption was verified based on Schoenfeld residuals.^20^

### Parametric time-to-event (TTE) model for OS

Further, parametric TTE modelling of OS was carried out using NONMEM 7.5.1 through Perl Speaks NONMEM (PSN 5.3.0) (ICON Early Phase, Dublin, Ireland) (Supplementary Material, Equation S1, available at https://doi.org/10.1016/j.esmorw.2024.100069). To define the baseline hazard h_0_(t), hazard functions of exponential, log logistic, Weibull and Gompertz distributions (Supplementary Material, Equation S2-S5, available at https://doi.org/10.1016/j.esmorw.2024.100069) were fitted to the data and the best fitting model evaluated based on objective function values (OFV) and visual predictive checks (VPC) using vpc, xpose and xpose4 packages in R. Then, the model was built with all the factors in the multivariable coxph model included as covariates. Survival estimates were then derived from the hazard estimates based on the relationship between survival and cumulative hazards (Supplementary Material, Equation S6, available at https://doi.org/10.1016/j.esmorw.2024.100069). A stratified VPC using 1000 simulations was generated to check for goodness of fit. Model performance was assessed based on the calibration plot.

## Results

### Cohort characteristics

**Figure 1:**
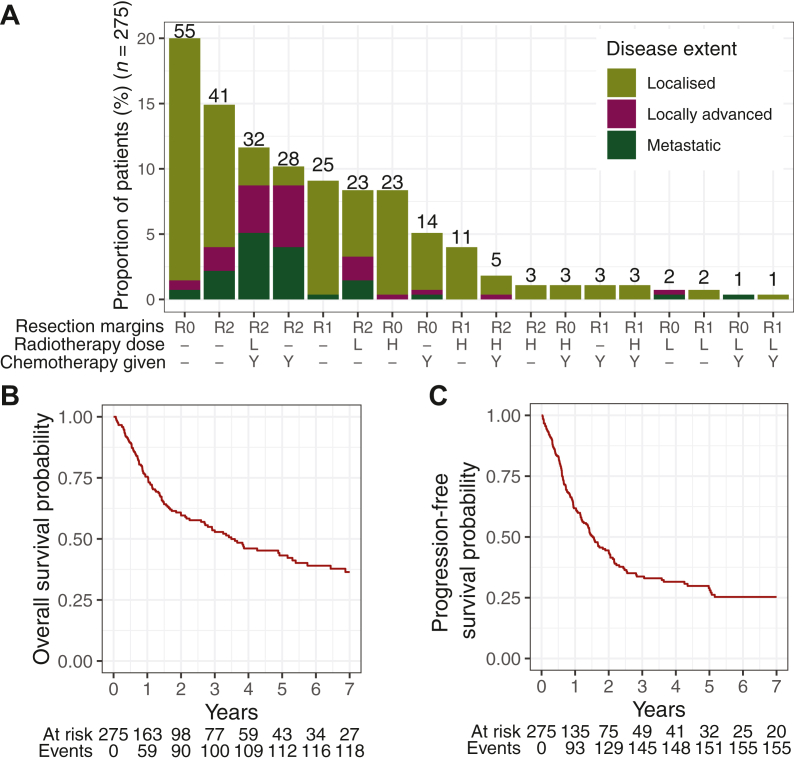
**Distribution of treatment modalities and survival outcomes of HNS patients.** (A) Frequency of treatment received by disease extent. Resection margins: R0 = microscopic margin clear, R1 = microscopic margin positive, R2 = R2 or no resection. Radiotherapy dose: − = not given, H = high dose, L = low dose. Chemotherapy given: − = not given, Y = given. Kaplan–Meier curves for (B) overall survival and (C) progression-free survival. HNS, head and neck sarcomas.

Out of the 286 patients classified to have HNS in the database, 275 met all eligibility criteria and were included in this study. Median age was 58 years (interquartile range: 38.5-71.5 years), with 44.4% of patients being female. The majority (73.5%) were Chinese, while Indians and Malays each made up 5.8%. The remaining 11.5% consisted of other minority races, mostly from regional Southeast Asia. A breakdown of sarcoma subtypes, disease extent and the interventions given to these patients are listed in Table 1. The most common treatment modality was monotherapy of wide excision with tumour-free microscopic margins (R0: 20%), followed by ‘R2 or no resection’ which included patients with gross residual tumour following treatment (14.9%). A combination of ‘R2 or no resection’ with chemotherapy and low-dose radiotherapy was the third most common (11.6%) (Figure 1A).

**Table 1:** Key cohort characteristics

Characteristics	All patients (*N* = 275)	
	*N*	%
Patient factors		
Age,^a^ years	58 (38.5-71.5)	
Sex		
Male	153	55.6
Female	122	44.4
Race		
Chinese	202	73.5
Indian	16	5.8
Malay	16	5.8
Others	41	14.9
Disease factors		
Disease extent		
Locally advanced	39	14.2
Localised	195	70.9
Metastatic	41	14.9
Radiotherapy-associated disease		
No	244	88.7
Yes	31	11.3
Histology		
Angiosarcoma	91	33.1
Rhabdomyosarcoma	33	12.0
UPS^b^	24	8.7
Others^c^	127	46.2
Treatment factors		
Resection margins		
R0	98	35.6
R1	45	16.4
R2 or no resection	132	48.0
Chemotherapy given		
Yes	90	32.7
No	185	67.3
Radiotherapy dose		
High	48	17.5
Low	61	22.2
None	166	60.8

a Data presented is median (interquartile range).

b Undifferentiated pleomorphic sarcoma (previously known as malignant fibrous histiocytoma).

c Low-frequency histological subtypes under others: alveolar soft-part sarcoma, chordoma, dermatofibrosarcoma protruberans, desmoid fibromatosis, fibrosarcoma, hemangiopericytoma/solitary fibrous tumour, Kaposi sarcoma, left biphenotypic sinonasal sarcoma, leiomyosarcoma, malignant peripheral nerve sheath tumour, myxofibrosarcoma, osteosarcoma, sarcoma—high-grade, sarcoma—NOS (not otherwise specified), spindle cell tumour, synovial sarcoma, tenosynovial diffuse type giant cell tumour, undifferentiated sarcoma—unspecified, Ewing’s sarcoma, liposarcoma.

Median follow-up was 3.42 years [95% confidence interval (CI) 2.16-4.29 years]. The cohort had a median of 3.49 years (95% CI 2.7-5.3 years) for OS (Figure 1B) and 1.52 years (95% CI 1.26-2.03 years) for PFS (Figure 1C). The 5-year OS was 43.2 % (95% CI 36.2% to 51.6%) and 5-year PFS was 28.9% (95% CI 22.9% to 36.6%).

### Age and disease extent are significant prognostic factors

**Figure 2:**
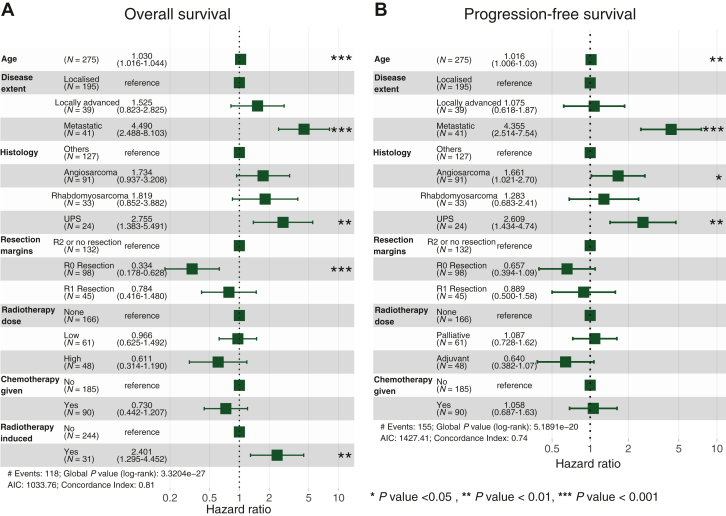
**Forestplot of clinically and statistically significant prognostic factors for overall survival (OS) and progression-free survival (PFS) using multivariable Cox proportional hazard (coxph) regression.** (A) Older age, metastatic disease at diagnosis, R0 surgical resection margins and radiotherapy-associated disease and the undifferentiated pleiomorphic sarcoma (UPS) subtype were associated with poorer OS. (B) Older age and metastatic disease at diagnosis were key adverse prognostic factors for PFS. Radiotherapy-associated disease was not included in the PFS model as it was not statistically significant on univariable analysis. Akaike's information criterion (AIC) was used to evaluate the model's goodness of fit.

Multivariable coxph analysis identified age [hazard ratio (HR) 1.03 per year difference from the median 58 years old (95% CI 1.02-1.04)], presence of metastasis at diagnosis [HR 4.49 (95% CI 2.49-8.10)] and radiotherapy-associated disease [HR 2.40 (95% CI 1.30-4.45)] as independent prognostic factors for OS (Figure 2A). For PFS, age [HR 1.02 (95% CI 1.01-1.03)] and presence of metastasis at diagnosis [HR 4.36 (95% CI 2.51-7.54)] were identified as prognostic factors (Figure 2B).

Among histologies, only Undifferentiated pleiomorphic sarcoma (UPS) was significant compared to other subtypes in OS, [HR 2.76 (95% CI 1.38-5.49)]. Angiosarcoma and rhabdomyosarcoma also showed higher HRs, but were not statistically significant. With PFS, both angiosarcoma [HR 1.66 (95% CI 1.02-2.70)] and UPS [HR 2.61 (95% CI 1.43-4.74)] were significant. This points towards these three subtypes being more aggressive compared to other sarcoma subtypes (Figure 2A and B).

### The effect of surgical resection margins, radiotherapy and chemotherapy on outcomes

Surgical resection was the most critical intervention associated with survival outcomes, with resection margins being the only treatment factor identified in our analysis to be significantly associated with improved outcomes. Compared with ‘R2 or no resection’, an R0 resection was significantly associated with improved OS [HR 0.33 (95% CI 0.18-0.63)], but not PFS [HR 0.66 (95% CI 0.39-1.09)]. The improvement in outcomes associated with an R1 resection was, however, not significant for OS [HR 0.78 (95% CI 0.42-1.48)] or PFS [HR 0.890 (95% CI 0.50-1.58)].

There were no distinct differences in OS [HR 0.97 (95% CI 0.63-1.49)] or PFS [HR 1.09 (95% CI 0.73-1.62)] between patients who did not receive radiotherapy and those who had low-dose radiotherapy. Compared with patients who did not receive radiotherapy, those who had high-dose radiotherapy had higher OS [HR 0.61 (95% CI 0.31-1.19)] and PFS [HR 0.64 (95% CI 0.38-1.07)], though these differences were not statistically significant.

Improvement in OS associated with chemotherapy use was not significant [HR 0.73 (95% CI 0.44-1.21)], and there was no significant association between chemotherapy use with PFS [HR 1.06 (95% CI 0.69-1.63)].

### Development of Head and Neck Sarcoma Assessor (HaNSA)—a parametric TTE model for HNS OS

To evaluate whether our multivariable coxph model could be used for simulations, we tested our model for proportional hazards. However, the proportional hazards test^20^ showed a global *P* value of 0.027, indicating that the baseline hazard would need to be defined before simulation studies. We therefore built our prognostic predictive tool based on a parametric TTE model which allowed us a robust method of testing various distributions to define a baseline hazard function. The log logistic distribution had the best fit for the baseline hazard function (OFV 595.7), compared to Weibull (OFV 618.9), exponential (OFV 624.9) and Gompertz (OFV 626.6), which led us to use the log logistic distribution as the base model for subsequent covariate modelling. Parameter estimates of the fitted full model are listed in Table 2. HR estimates of the various covariates were largely similar with those based on the coxph model. VPC on the predicted model estimates against observed data indicated a good model fit and performance (Supplementary Figure S1, available at https://doi.org/10.1016/j.esmorw.2024.100069).

**Table 2:** Parametric time-to-event (TTE) model parameters

Parameter category	Parameter	pTTE estimate (RSE)	pTTE HR	Coxph HR
Baseline hazard	λ	0.342 (0.329)		
	γ	1.61 (0.067)		
Age	Age	0.016 (0.160)	1.02	1.03
Radiotherapy-associated	No	Reference		
	Yes	0.786 (0.561)	2.19	2.4
Resection margins	R2 or no resection	Reference		
	R0 resection	−0.680 (0.138)	0.51	0.33
	R1 resection	−0.286 (0.840)	0.75	0.78
Disease extent	Localised	Reference		
	Locally advanced	0.211 (1.56)	1.23	1.53
	Metastatic	2.50 (0.410)	12.18	4.49
Histology	Others	Reference		
	Angiosarcoma	0.758 (0.608)	2.13	1.73
	Rhabdomyosarcoma	0.590 (0.865)	1.80	1.82
	Undifferentiated pleiomorphic sarcoma	1.484 (0.517)	4.41	2.76
Radiotherapy dose	None	Reference		
	High	−0.390 (0.464)	0.68	0.61
	Low	−0.075 (2.93)	0.93	0.97
Chemotherapy given	No	Reference		
	Yes	−0.223 (0.771)	0.80	0.73

Coxph, Cox proportional hazard; HR, hazard ratio; pTTE, parametric TTE; RSE, relative standard error.

### Personalised prognosis predictions using HaNSA

Using the final parametric TTE model for OS, we simulated OS for all possible combinations of covariate values in the model, enabling clinicians to evaluate and visualise the impact of different interventions on OS. We made the simulations available as a browser tool using RShiny (HaNSA) to help clinicians evaluate and visualise the impact of different interventions on OS: https://research-data-integration.shinyapps.io/HeadAndNeckSarcomaAssessor/.

**Figure 3:**
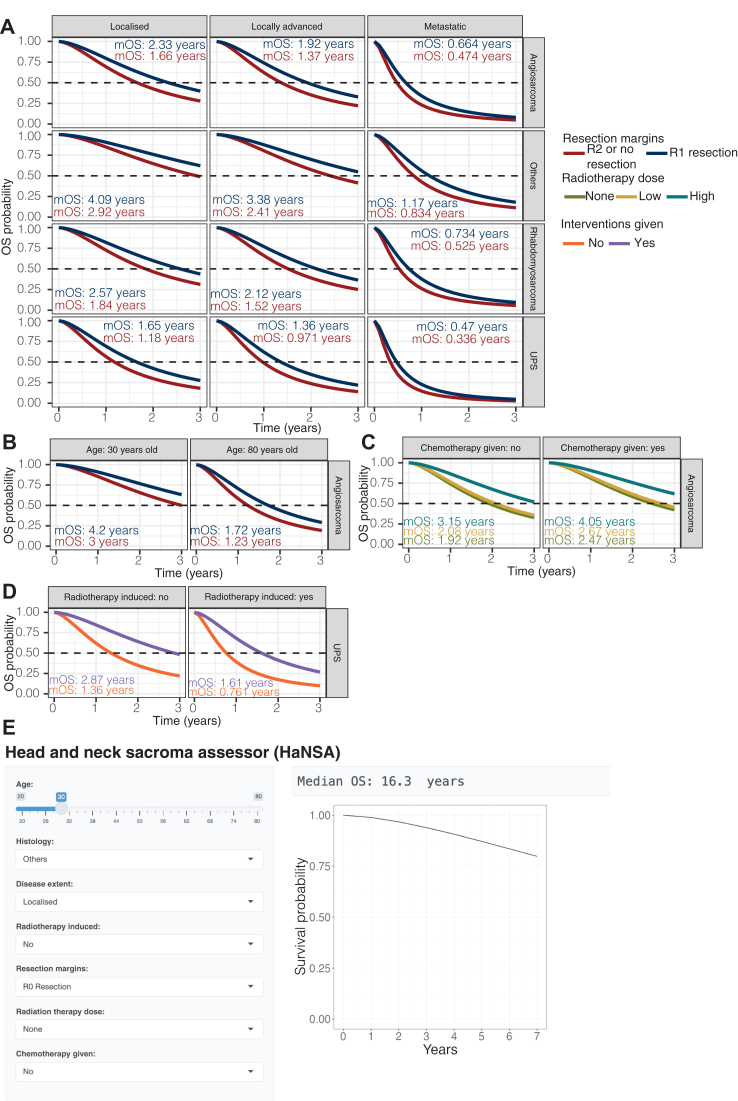
**Predictedoverall survival (OS) for different clinical scenarios simulated with a parametric time-to-event model.** (A) Comparison of predicted OS in R1 resection versus ‘R2 or no resection’ across different sarcoma subtypes and disease extent in a 58-year-old patient (median age of study cohort) with no radiotherapy-associated sarcoma and who did not receive chemotherapy. R1 resections had higher median OS across the board than ‘R2 or no resection’, especially in localised and locally advanced disease extent. (B) Comparison of predicted OS in R1 resections versus ‘R2 or no resection’ in a 30-year-old and an 80-year-old patient, each with localised angiosarcoma which was not radiotherapy associated and not given chemotherapy. Compared with ‘R2 or no resection’, median OS of R1 resection was higher by 1.2 years for the 30-year-old patient and 0.49 years for the 80-year-old patient. (C) Comparison of predicted OS treated with different radiotherapy doses, with and without chemotherapy in a 58-year-old patient with locally advanced angiosarcoma and R1 resection margins. A high dose of radiotherapy is required for significant OS benefit with or without chemotherapy. (D) Comparison of predicted OS in radiotherapy-associated versus not radiotherapy-associated angiosarcoma in a 58-year-old patient that is locally advanced, with and without all key interventions. While having all key interventions can improve median OS by a year in the radiotherapy-associated simulation, the outcome remains dismal. (E) The RShiny app interface that allows clinicians to input patient and disease factors, as well as planned treatments to predict OS.

To illustrate the utility of the tool, we simulated four different clinical scenarios to access the benefits of R1 resections compared to R2 or no resections, since R1 resections are common with R0 resections being anatomically difficult to achieve. The four clinical scenarios were as follows: (i) R1 resections in different subtypes and disease extents (Figure 3A), (ii) young versus old patients with R1 resections for angiosarcoma (Figure 3B), (iii) the effect of radiotherapy and chemotherapy in combination with R1 resection (Figure 3C) and (iv) the effect of all interventions in combination on sarcomas that were or were not radiotherapy associated for UPS (Figure 3D). The web application interface is shown in Figure 3E.

Firstly, R1 resection increased median OS (mOS) over a wide range from 0.134 to 1.17 years, compared to no resection. Angiosarcoma, rhabdomyosarcoma and UPS as more aggressive subtypes had smaller OS benefit, with UPS showing the least benefit (mOS increase <0.5 years). With metastatic disease, little benefit in OS (mOS increase <0.5 years) was similarly observed. Secondly, we investigated the impact of age alongside an R1 resection. For a 30-year-old patient, R1 resection increased mOS by 1.2 years, while in an 80-year-old, the benefit was much less at 0.49 years increase in mOS (Figure 3B). The mOS in the 30-year-old was notably higher at 4.2 years, compared to 1.72 years in the 80-year-old, when R1 resection was carried out (Figure 3B). Thirdly, we explored the treatments in combination with R1 resection. High-dose radiotherapy showed the most benefit, increasing mOS by 1.23 years without chemotherapy and 1.58 years with chemotherapy (Figure 3C). Low-dose radiotherapy, on the other hand, did not significantly change OS. Adding chemotherapy alongside high-dose radiotherapy showed the most benefit in OS, improving mOS by 0.9 years compared to high-dose radiotherapy alone. Lastly, we studied the impact of giving all key interventions on radiotherapy-associated sarcoma. Even when all interventions were given, patients with radiotherapy-associated sarcomas still had a 1.26-year lower mOS compared to patients whose sarcomas were not radiotherapy induced, highlighting the aggressiveness of the disease. Together, these clinical simulations serve to show how HaNSA can be used as a versatile prognostic tool to evaluate the impact of different treatment interventions on OS with specific disease and patient factors.

## Discussion

This is the largest HNS study for a multi-ethnic Asian population to date. We chose to focus specifically on HNS for their distinct biology, unique treatment constraints and outcomes poorer than sarcomas as a whole.^3^ Globally, HNS data are scattered with differing pathological interpretations of histologies from non-sarcoma-trained pathologists. In fact, most centres do not provide granularity of histological subtype beyond a general ‘sarcoma’ diagnosis. It is therefore unsurprising that most centres capture few patients on record.^21^ Building on previous analyses, a large cohort for HNS of 275 patients and accompanying matched patient prognostic factors such as age, disease extent, histology and treatment interventions of surgical resection, radiotherapy and chemotherapy were curated. While age and disease extent were significant prognostic factors for both OS and PFS, radiotherapy-associated disease was prognostically significant only for OS. Only R0 resection margins were statistically significant in improving OS among interventions. Using parametric TTE modelling, we developed HaNSA, a prognostic calculator which incorporates patient and disease factors, as well as commonly employed interventions in HNS to provide a holistic projection of a patient’s prognosis based on their presentation and treatment plan.

Our cohort had a lower 5-year OS (43.2%), compared to an equivalent larger Western HNS study (5-year OS: Peng et al. 66%^7^^,^^22^^,^^23^), highlighting a more aggressive phenotype in our population. We highlighted that age and disease extent are significant prognostic factors for both OS and PFS. This has been evaluated in other studies on HNS^24^^,^^25^ and other cancer types.^26^, ^27^, ^28^ For example, Chang et al.^24^ reported similar findings where older HNS patients with metastatic disease performed significantly worse. Notably, our cohort had a 75-fold higher incidence of radiotherapy-associated sarcoma at 11.3% (Table 1), compared to 0.15% in global meta-analysis.^29^ This could be due to a high rate of nasopharyngeal carcinoma (NPC) in Singapore,^30^ where high-dose radiotherapy to the head and neck region is the key treatment.^31^ A Taiwanese study with high rates of NPC similarly reported a high rate of radiotherapy-associated sarcoma.^32^ Interestingly, while radiotherapy-associated sarcoma was significantly associated with worse OS similar to other studies,^29^^,^^32^ there was no significant association with PFS. This was observed as patients with radiotherapy-associated sarcomas had very close PFS and OS intervals (Supplementary Figure S2, available at https://doi.org/10.1016/j.esmorw.2024.100069), suggesting they did not survive for long after disease progression. Closer follow-up for NPC patients might be warranted to detect radiotherapy-associated sarcomas early, in order to better treat these patients and improve OS.

Not all histological subtypes significantly impacted OS. Interestingly, while angiosarcoma and rhabdomyosarcoma are often labelled as aggressive subtypes, these subtype differences were not significant after accounting for treatment modalities and disease extent. Only UPS was statistically significant for both OS and PFS. This suggests that early disease detection and selecting the right treatment modality appropriate for the histology subtype could be more important in improving OS than the subtype itself being deterministic in prognosis. Development of better treatments for UPS is thus even more urgent compared to other subtypes, however, as this subtype showed a significantly poorer OS.

With interventions, only surgical resections were significantly associated with OS. However, we chose to keep chemotherapy and radiotherapy in the model as these were still important commonly used clinical interventions.^33^, ^34^, ^35^, ^36^, ^37^ While certain anatomic sites are more challenging to resect, ultimately what determines their prognosis is the surgical resection margin, rather than the site itself. Even though univariate Cox showed site as a significant category, in a multivariate Cox with both site and resection margins, we found that site was no longer statistically significant (Supplementary Figure S3, available at https://doi.org/10.1016/j.esmorw.2024.100069). We thus chose histology and resection margins instead of including the site of primary tumour as covariates in our model. HaNSA further shows how treatments can compound together to improve OS, compared to single treatments alone, highlighting the importance of considering all common treatment interventions in a prognostic calculator.

Unlike other sarcoma prognostic calculators which were built on larger datasets (Sarculator 9738 patients,^17^ PERSARC 766 patients^18^), our HNS study (275 patients), while being a large cohort for HNS, was significantly smaller. The use of parametric TTE alongside VPC for diagnostic checks was thus more suited for this study compared to classical machine learning methods which rely heavily on big/homogenous cohorts for good accuracy.^38^ Using VPC, we showed good model fit even when the data were stratified across all 18 possible covariates (Supplementary Figure S1, available at https://doi.org/10.1016/j.esmorw.2024.100069).^39^ We were also able to test multiple different baseline hazard distributions (exponential, Weibull, Gompertz and log logistic) to find the best fit for the baseline hazard to derive survival estimates, whereas multivariable Cox regression model with an undefined baseline hazard distribution commonly adopts empirical survival estimation using either the Breslow or the Kalbfleisch and Prentice method.^40^ The use of parametric TTE is thus prudent for HNS as a rare disease, where large numbers are not often achievable.

Furthermore, our prognostic calculator evaluates the survival outcome of each key intervention and is the first of its kind for HNS. Other prognostic calculators, e.g. Sarculator [retroperitoneal and extremities soft-tissue sarcoma (eSTS)]^17^ and PERSARC (eSTS),^18^ do not describe HNS or Asian populations. Sarculator also accounts only for patient demographics and disease severity, but only surgical resection for treatment. Meanwhile, HaNSA allows us to evaluate the survival outcome of each key intervention both singly and in combination, while also accounting for different patient ages and disease presentations.

However, our model focuses mainly on quantifying OS. Holistic patient care which takes into consideration intervention impact on symptom palliation and quality of life should still be taken into account by the clinician before deciding on the final treatment.

### Limitations

Our study faced several limitations. Firstly, the database spanned across 30 years and we are unable to interrogate every factor that might have contributed to a gradual improvement in treatment outcome over time. While there have been no landmark advances in therapeutics, gradual improvements in diagnosis, management and supportive care over the years might have contributed to better OS.^41^ Secondly, our cohort was from a single centre which may impede the generalisability of the findings. Independent validation using a separate cohort is required. Lastly, as a retrospective cohort study, we could only evaluate associations on patient outcomes and not determine any actual cause–effect relationship.^42^ Future large-scale prospective cohort studies are imperative to guide clinical decisions to better support this vulnerable group.

### Conclusion

In this study, we demonstrate a new paradigm as to how meaningfully curated real-world data in a rare disease entity can be used to predict the OS of individual patients with specific treatment approaches. Our analysis provides key insights into a previously poorly described Asian population, and our prognostic predictive tool can guide HNS therapy decision making. Future validation with external cohorts (as they become available) and adaptations to involve other patient populations and cohorts are essential.

## References

[1] Sharifnia T., Hong A.L., Painter C.A., Boehm J.S. (2017). Emerging opportunities for target discovery in rare cancers. Cell Chem Biol.

[2] Low C.E., Loke S., Pang G.E., Sim B., Yang V.S. (2024). Psychological outcomes in patients with rare cancers: a systematic review and meta-analysis. eClinicalMedicine.

[3] Kalavrezos N., Sinha D. (2020). Head and neck sarcomas in adulthood: current trends and evolving management concepts. Br J Oral Maxillofac Surg.

[4] Zagars G.K., Ballo M.T., Pisters P.W.T. (2003). Prognostic factors for patients with localized soft-tissue sarcoma treated with conservation surgery and radiation therapy: an analysis of 1225 patients. Cancer.

[5] Kassir R.R., Rassekh C.H., Kinsella J.B., Segas J., Carrau R.L., Hokanson J.A. (1997). Osteosarcoma of the head and neck: meta-analysis of nonrandomized studies. Laryngoscope.

[6] Mattavelli D., Miceli R., Radaelli S. (2013). Head and neck soft tissue sarcomas: prognostic factors and outcome in a series of patients treated at a single institution. Ann Oncol.

[7] Peng K.A., Grogan T., Wang M.B. (2014). Head and neck sarcomas: analysis of the SEER database. Otolaryngol Head Neck Surg.

[8] Park J.T., Roh J.L., Kim S.O. (2015). Prognostic factors and oncological outcomes of 122 head and neck soft tissue sarcoma patients treated at a single institution. Ann Surg Oncol.

[9] González-González R., Bologna-Molina R., Molina-Frechero N., Domínguez-Malagon H.R. (2012). Prognostic factors and treatment strategies for adult head and neck soft tissue sarcoma. Int J Oral Maxillofac Surg.

[10] Yanzon A., Gomez N.L., Picco P. (2021). Head and neck sarcomas: treatment outcomes in a tertiary referral center in Argentina. Oral Maxillofac Surg.

[11] Cannon R.B., Kull A.J., Carpenter P.S. (2019). Adjuvant radiation for positive margins in adult head and neck sarcomas is associated with improved survival: analysis of the National Cancer Database. Head Neck.

[12] Tajudeen B.A., Fuller J., Lai C. (2014). Head and neck sarcomas: the UCLA experience. Am J Otolaryngol.

[13] Salcedo-Hernández R.A., Lino-Silva L.S., Mosqueda-Taylor A., Luna-Ortiz K. (2014). Soft tissue sarcomas of the head and neck. Clinical and pathological evaluation of 108 cases in Mexico. J Craniomaxillofac Surg.

[14] Galy-Bernadoy C., Garrel R. (2016). Head and neck soft-tissue sarcoma in adults. Eur Ann Otorhinolaryngol Head Neck Dis.

[15] Sabesan T., Xuexi W., Yongfa Q., Pingzhang T., Ilankovan V. (2006). Malignant fibrous histiocytoma: outcome of tumours in the head and neck compared with those in the trunk and extremities. Br J Oral Maxillofac Surg.

[16] Edge S.B., Compton C.C. (2010). The American Joint Committee on Cancer: the 7th edition of the AJCC cancer staging manual and the future of TNM. Ann Surg Oncol.

[17] Voss R.K., Callegaro D., Chiang Y.J. (2022). Sarculator is a good model to predict survival in resected extremity and trunk sarcomas in US patients. Ann Surg Oncol.

[18] van Praag V.M., Rueten-Budde A.J., Jeys L.M. (2017). A prediction model for treatment decisions in high-grade extremity soft-tissue sarcomas: personalised sarcoma care (PERSARC). Eur J Cancer.

[19] Chen Y., Shen Q., Gokavarapu S. (2016). Osteosarcoma of head and neck: a retrospective study on prognostic factors from a single institute database. Oral Oncol.

[20] Grambsch P.M., Therneau T.M. (1994). Proportional hazards tests and diagnostics based on weighted residuals. Biometrika.

[21] Florou V., Nascimento A.G., Gulia A., de Lima Lopes G. (2018). Global health perspective in sarcomas and other rare cancers. Am Soc Clin Oncol Educ Book.

[22] Yeang M.S., Tay K., Ong W.S. (2013). Outcomes and prognostic factors of post-irradiation and de novo sarcomas of the head and neck: a histologically matched case-control study. Ann Surg Oncol.

[23] Koh Y.S., Chan J.Y., Looi W.S. (2022). Outcomes of head and neck angiosarcoma with different treatment modalities: a 20-year single institutional experience. Precis Cancer Med.

[24] Chang A.E., Chai X., Pollack S.M. (2014). Analysis of clinical prognostic factors for adult patients with head and neck sarcomas. Otolaryngol Neck Surg.

[25] Pisani P., Airoldi M., Allais A. (2020). Metastatic disease in head & neck oncology. Acta Otorhinolaryngol Ital.

[26] White M.C., Holman D.M., Boehm J.E., Peipins L.A., Grossman M., Jane Henley S. (2014). Age and cancer risk. Am J Prev Med.

[27] Hao Y., Li G. (2023). Prediction of distant organ metastasis and overall survival of lung cancer patients: a SEER population−based cohort study. Front Oncol.

[28] Low C.E., Yau C.E., Tan R.Y. (2024). Association of depression with all-cause and cancer-specific mortality in older adults with cancer: systematic review, meta-analysis, and meta-regression. J Geriatr Oncol.

[29] Coca-Pelaz A., Mäkitie A.A., Strojan P. (2021). Radiation-induced sarcomas of the head and neck: a systematic review. Adv Ther.

[30] (2024). HealthHub. Nasopharyngeal-Cancer. https://www.healthhub.sg/a-z/diseases-and-conditions/nasopharyngeal-cancer.

[31] Jicman Stan D., Niculet E., Lungu M. (2022). Nasopharyngeal carcinoma: a new synthesis of literature data (review). Exp Ther Med.

[32] Chen W.Y., Lu S.H., Wang Y.M. (2023). Post-irradiation sarcoma after definitive radiation therapy for nasopharyngeal carcinoma. Radiother Oncol.

[33] Khadembaschi D., Jafri M., Praveen P., Parmar S., Breik O. (2022). Does neoadjuvant chemotherapy provide a survival benefit in maxillofacial osteosarcoma: a systematic review and pooled analysis. Oral Oncol.

[34] Tudor-Green B., Fonseca F.P., Gomez R.S., Brennan P.A. (2017). Current update on the diagnosis and management of head and neck hard tissue sarcomas. J Oral Pathol Med.

[35] König M., Osnes T.A., Lobmaier I. (2017). Multimodal treatment of craniofacial osteosarcoma with high-grade histology. a single-center experience over 35 years. Neurosurg Rev.

[36] Sun H., Liu J., Hu F. (2023). Current research and management of undifferentiated pleomorphic sarcoma/myofibrosarcoma. Front Genet.

[37] O’steen L., Saldivar B., Kharod S., Bassett B., Morris C.G., Mendenhall W.M. (2020). Radiotherapy for adult soft tissue sarcomas of the head and neck. Am J Clin Oncol.

[38] Cascarano A., Mur-Petit J., Hernández-González J. (2023). Machine and deep learning for longitudinal biomedical data: a review of methods and applications. Artif Intell Rev.

[39] Holford N. (2013). A time to event tutorial for pharmacometricians. CPT Pharmacomet Syst Pharmacol.

[40] Xia F., Ning J., Huang X. (2018). Empirical comparison of the Breslow estimator and the Kalbfleisch Prentice estimator for survival functions. J Biom Biostat.

[41] Low C.E., Pillay R.M., Teo F.J.J. (2024). Educational interventions to reduce depression and anxiety in older adults with cancer in the community: a systematic review, meta-analysis and meta-regression of randomised controlled trials. Age Ageing.

[42] Low C.E., Teo Y.N., Teo Y.H. (2023). Propensity-score matched analysis of patent foramen ovale closure in real-world study cohort with cryptogenic ischemic stroke. J Stroke Cerebrovasc Dis.

