# Supplementary material for: Head and Neck Sarcoma Assessor (HaNSA) for treatment decisions using real-world data
Source: ESMO Real World Data Digit Oncol. 2024 Sep 5;5:100069. doi: 10.1016/j.esmorw.2024.100069 (PMC12836604; doi:10.1016/j.esmorw.2024.100069)
Supplement: Supplementary data [file mmc1.docx]

**Head and Neck Sarcoma Assessor (HaNSA) for treatment decisions using real-world data - Supplementary material**

Final parametric model with covariates included


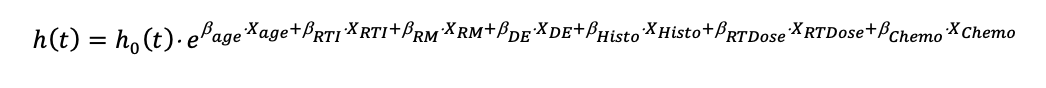
SEquation 1.

h(t) = hazard at time t,

h_0_(t) = baseline hazard at time t,

β_age_= covariate parameter for age,

X_age_ = Patient age - median age,

β_RTI_ = covariate parameter for radiotherapy induced disease,

X_RTI_ = Radiotherapy induced disease (No = 0, Yes = 1),

β_RM_ = covariate parameter for corresponding resection margin category,

X_RM_ = Resection margins (R2 or no resection = 0, R1 resection = 1, R0 resection = 1),

β_DE_ = covariate parameter for corresponding disease extent category,

X_DE_ = Disease extent (Localised = 0, Locally advanced = 1, Metastasis = 1),

β_Histo_ = covariate parameter for corresponding histology,

X_Histo_ = Histology (Others = 0, Angiosarcoma = 1, Rhabdomyosarcoma = 1, UPS/MFH = 1),

β_RTDose_ = covariate parameter for corresponding radiotherapy dose,

X_RTDose_ = Radiotherapy dose (None = 0, Low = 1, High = 1),

β_Chemo_ = covariate parameter for chemotherapy given,

X_Chemo_ = Chemotherapy given (No = 0 ,Yes = 1)

Baseline hazard was described using the hazard function of a log logistic distribution.


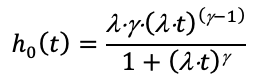
SEquation 2.

h_0_(t) = baseline hazard at time t, λ = scale parameter, γ = shape parameter, t = time

Alternative baseline hazards that were considered

Exponential


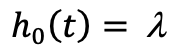
SEquation 3.

Gompertz


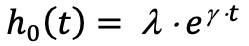
SEquation 4.

Weibull


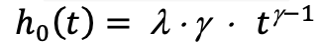
SEquation 5.

Survival probability was derived as the exponential of the cumulative hazard.


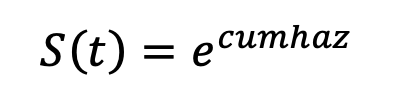


SEquation 6.

S(t) = survival probability, cumhaz = cumulative hazard, which is the integral of h(t)

eTable 1. Data dictionary

| **Variable** | **Description** | **Included in the final OS model?** |
| --- | --- | --- |
| **Outcome data** | | |
| Overall Survival (OS) (years) | Time from date of diagnosis to date of patient death, measured in years. Otherwise censored at last follow up or at 7 years, whichever occurred first | Yes |
| Progression free survival (PFS) (years) | Time from date of diagnosis to date of disease progression, recurrence, or death. Otherwise censored at last follow up or at 7 years, whichever occurred first | No |
| **Patient factors** | | |
| Age, years | Measured in years at the time of diagnosis | Yes |
| Sex | Categories used are:   1. Male 2. Female | No |
| Race | Categories used are:   1. Chinese 2. Indian 3. Malay 4. Others (not specified) | No |
| Eastern Cooperative Oncology Group (ECOG) | The ECOG Performance status scale describes a patient’s level of functioning in terms of their ability to care for themself, daily activity, and physical ability (walking, working, etc.)  The categories used are:   1. 0 2. 1 3. 2 4. 3 5. 4   *breakdown of ECOG gradings can be found online | No |
| **Disease factors** | | |
| Disease extent | As indicated in clinical notes - based on the judgement of the attending clinician following review of staging scans and other relevant investigations  The categories used are:   1. Localised 2. Locally advanced 3. Metastatic | Yes |
| Primary disease site | Defined as the area where the primary sarcoma originated from. Only sarcomas located at or above the level of the clavicles were included. Sarcomas originating within the cranial vault or spinal column were excluded.  The categories used are:   1. Bone (only bony sarcomas) 2. Upper aerodigestive tract (nasal vestibule and cavities, perinasal sinuses, oral cavity, pharynx, trachea and oesophagus) 3. Skin (of the head and neck, excluding the scalp) 4. Scalp (skin where hair grows, excluding facial hair) 5. Face, parotids, jaw (deep soft tissues of the head) 6. Neck (deep soft tissues of the neck) | No |
| Location of metastasis at diagnosis | As reported on staging scans  The categories used are:   1. None 2. Lymph nodes 3. Distant sites (including lungs, brain, liver and others) | No |
| Radiotherapy-induced disease | As indicated in clinical notes. Most commonly in patients previously treated for nasopharyngeal carcinoma or lymphoma of the head and neck.  The categories used are:   1. Yes 2. No | Yes |
| Histology | As confirmed after review by at least two senior pathologists  The categories used are:   1. Angiosarcoma (including Epitheloid hemangioendothelioma) 2. Rhabdomyosarcoma (including alveolar, embryonal, pleomorphic, spindle cell, unspecified 3. Osteosarcoma (including chondroblastic, conventional, extraosseous, osteoblastic, pleomorphic) 4. UPS/ MFH (undifferentiated pleomorphic sarcoma, previously known as malignant fibrous histiocytoma) 5. Others (including Alveolar soft part sarcoma, Chordoma, Dermatofibrosarcoma Protruberans, Desmoid/Fibromatosis, Fibrosarcoma, hemangiopericytoma/ Solitary Fibrous Tumor, Kaposi Sarcoma, Left Biphenotypic Sinonasal Sarcoma, Leiomyosarcoma, Malignant Peripheral Nerve Sheath Tumor, Myxofibrosarcoma, Osteosarcoma, Sarcoma - High Grade, Sarcoma - not otherwise specified, Spindle cell tumour, Synovial sarcoma, Tenosynovial Diffuse Type Giant Cell Tumour, Undifferentiated sarcoma - Unspecified)   *Subtypes were categorised as others due their scarcity | Yes |
| Grade | Based on Fédération Nationale des Centres de Lutte Contre le Cancer (FNCLCC).  The categories used are:   1. High (G3) 2. Moderate (G2) 3. Low (G1) | No |
| Presenting symptom | As identified from clinical notes  The categories used are:   1. Local (including mass effects, lumps and bleeding) 2. Systemic (including loss of weight or appetite, fever, metastatic symptoms such as bony aches or shortness of breath) 3. Both (local and systemic) 4. Incidental (findings during investigations for other reasons) | No |
| Size | As measured on radiological imaging, or estimated clinically  The categories used are:   1. <5cm 2. ≥5cm | No |
| Biomarkers | Samples of full blood counts and liver function tests taken at the date closest to the date of diagnosis were used.  The categories used are:   1. Neutrophil-to-lymphocyte ratio 2. Albumin-to-globulin ratio 3. Platelet-to-lymphocyte ratio | No |
| **Treatment Factors** | | |
| Resection margins | As confirmed by microscopic examination of surgical specimens after wide excision surgery  The categories used are:   1. R0 (Microscopic margin negative) 2. R1 (Microscopic margin positive) 3. R2 or no resection (gross tumour left behind) | Yes |
| Wide Excision surgery | The categories used are:   1. Yes 2. No (including biopsy only) | No |
| Chemotherapy given | The categories used are:   1. Yes 2. No | Yes |
| Chemotherapy intent | The categories used are:   1. Adjuvant (aiming for curative treatment) 2. Palliative (not aiming for curative treatment) 3. Not done | No |
| Chemotherapy Regime | The categories used are:   1. Combination 2. Single Agent 3. Not done | No |
| Neoadjuvant Chemotherapy Regime | The categories used are:   1. Combination 2. Single Agent 3. Not done | No |
| Radiotherapy Dose | The categories used are:   1. High dose (50-66Gy/25 to 33#) 2. Low dose (8 to 30 Gy in 1 to 10#) 3. None | Yes |
| Radiotherapy Intent | The categories used are:   1. Adjuvant (aiming for curative treatment) 2. Palliative (not aiming for curative treatment) 3. Not done | No |
| Radiotherapy site | The categories used are:   1. Local (targeting primary tumour) 2. Distant (targeting metastatic site) 3. Local & distant 4. None | No |

eTable 2: feature selection reasoning for each category of highly correlated parameters

| **Correlated parameters** | **Selected parameter** | **Reasoning** |
| --- | --- | --- |
| Wide excision, resection margins | Resection margins | Resection margins  Resection margins as it gives more granularity to the kind of resection done |
| Size,disease extent, N0N1, M1M0,  Lung mets, Bone mets | Disease extent | Disease extent(i.e. localised, locally advanced, metastatic) is all encompassing of the other 3 categories |
| Site, histology | Histology | Histology gives better insight to actual type of sarcoma being treated |


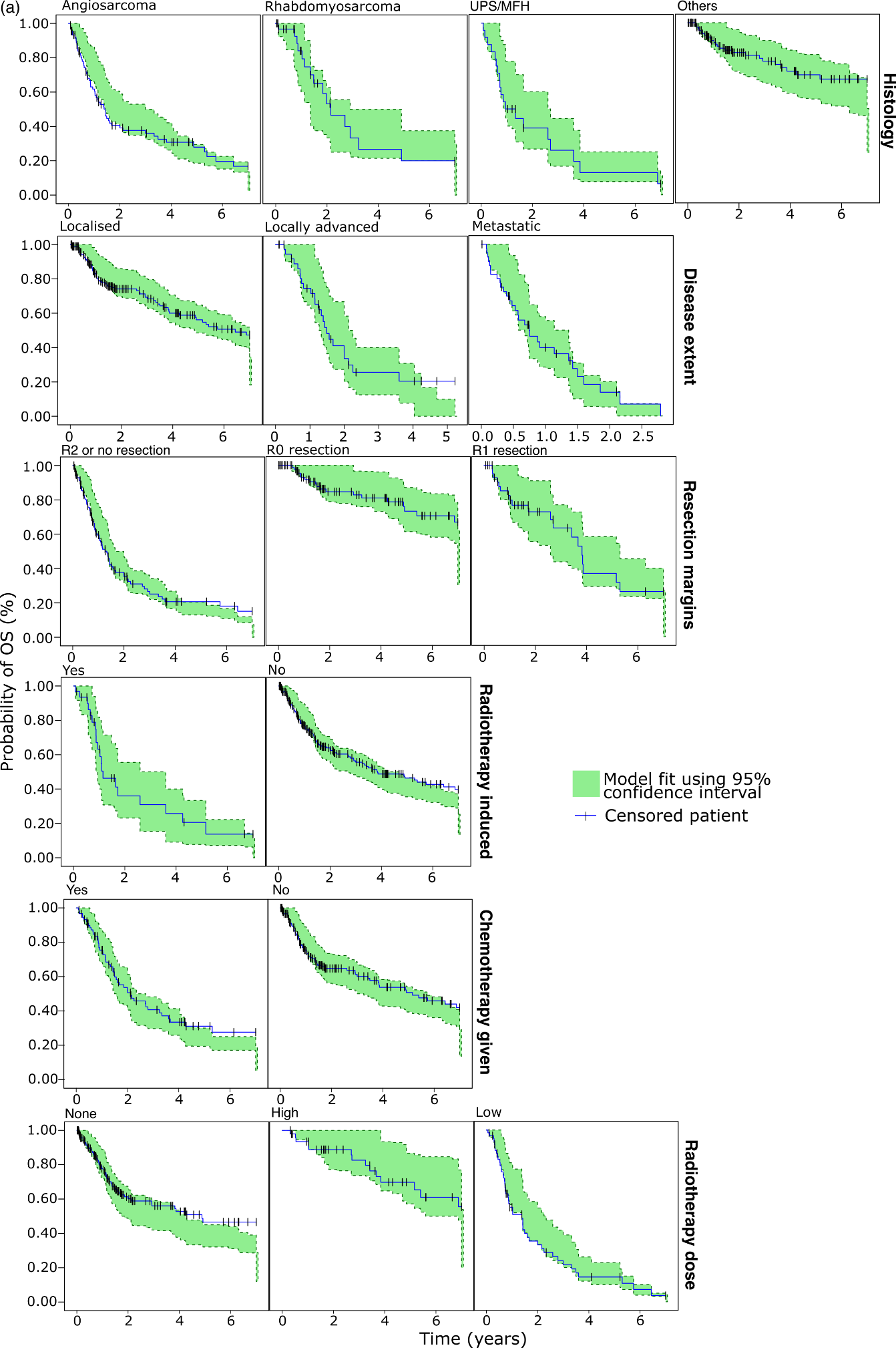


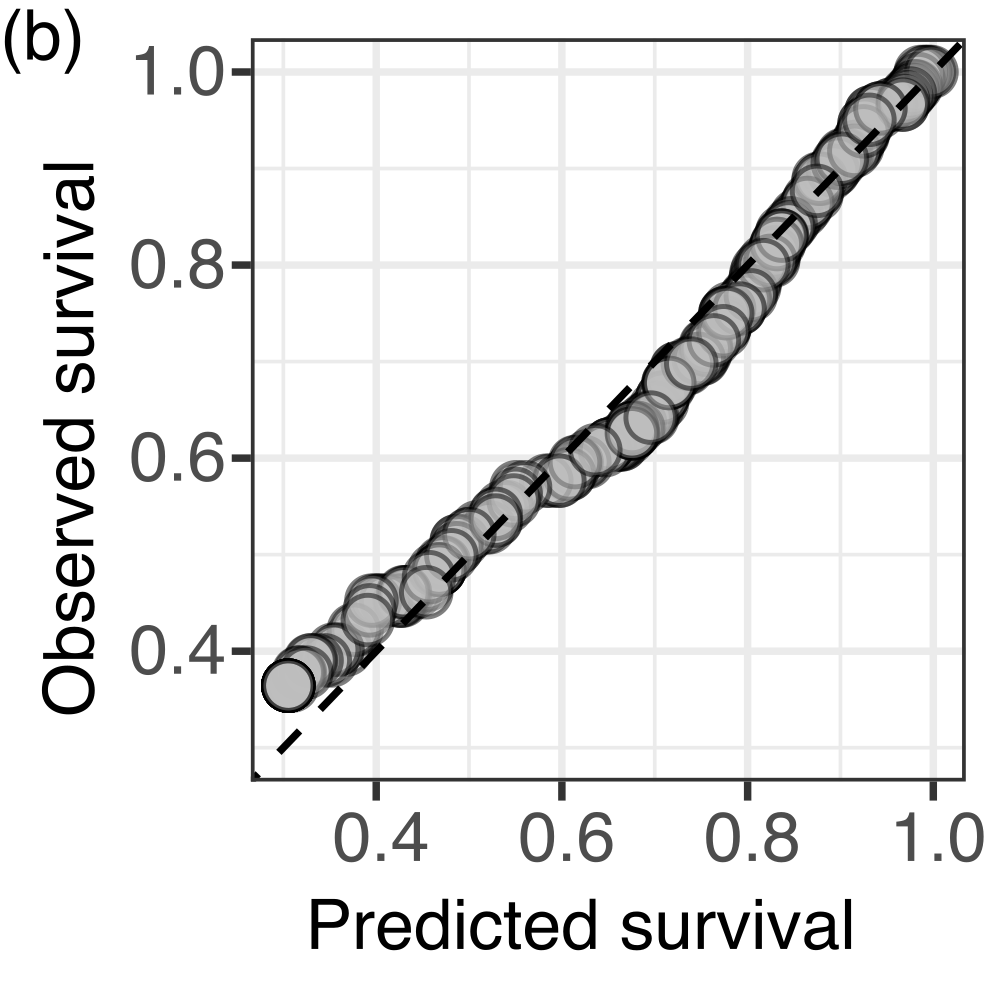


eFigure 1. Model diagnostics and performance of the fitted parametric time to event model for overall survival (OS). (a) Stratified visual predictive check plots show that the model fit was reasonable, with the Kaplan-Meier curve of OS lying within the 95% confidence band of predicted OS for each categorical prognostic factor included in the model. (b) Calibration plot shows the predicted OS were close to the observed OS for patients at different risk levels.


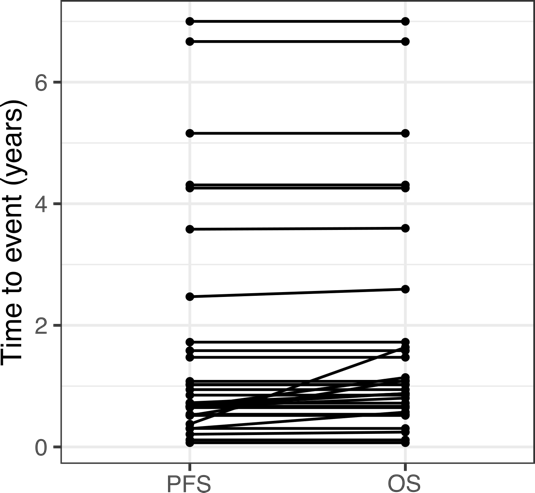


eFigure 2. A comparison of OS and PFS in all patients who had radiotherapy-induced sarcoma show little to no difference in duration, suggesting most patients passed on soon after progression.


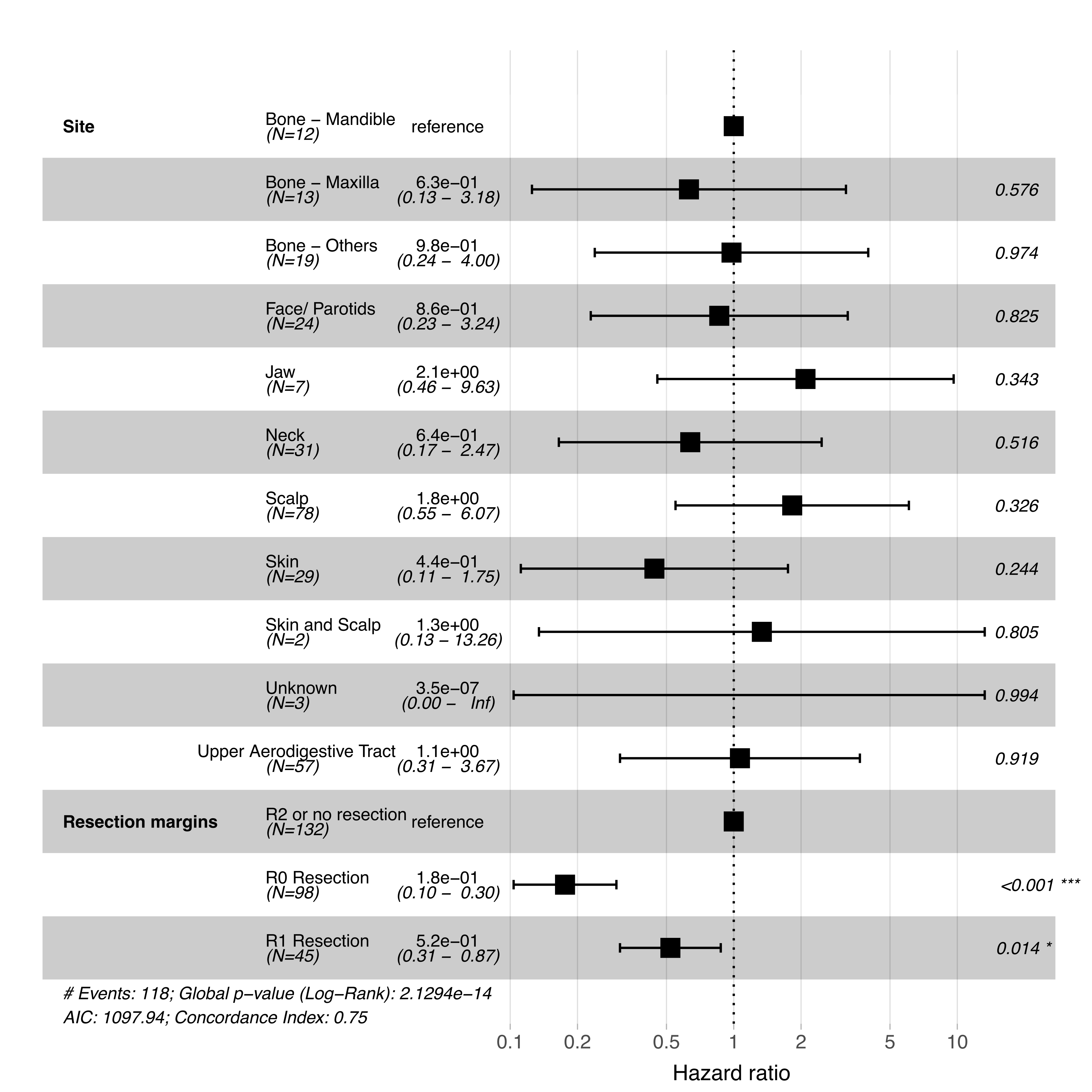
eFigure 3. Forrest plot of site and resection margin impact on overall survival. While univariate cox analysis suggested both categories might be important, upon adding both together in a multivariate analysis, site is no longer statistically significant.
